# Supplementary material for: The Small RNA Universe of Capitella teleta
Source: Front Mol Biosci. 2022 Feb 25;9:802814. doi: 10.3389/fmolb.2022.802814 (PMC8915122; doi:10.3389/fmolb.2022.802814)
Supplement: Supplementary file 1 [file DataSheet1.ZIP › Supplement/known/CAPTEscaffold_19_2484.pdf]

Provisional ID	:	CAPTEscaffold_19_2484
Score total	:	381098.4
Score for star read(s)	:	3.9
Score for read counts	:	381088.3
Score for mfe	:	1.6
Score for randfold	:	1.6
Score for cons. seed	:	3
Total read count	:	747500
Mature read count	:	715408
Loop read count	:	40
Star read count	:	32052

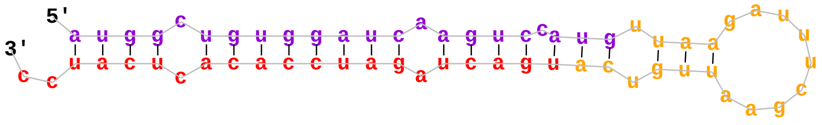

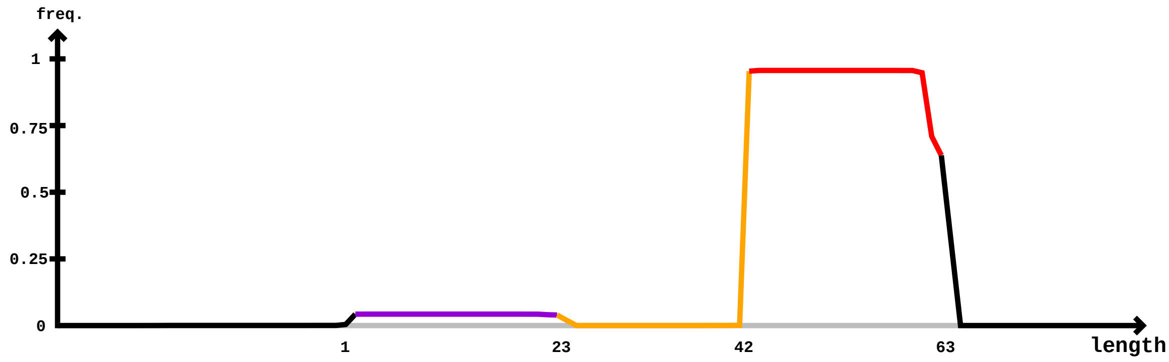

Star Mature

```
5'- gauuuugugucguuucgcugucgccuugaauggcuguggaucaaguccauguuaagauuucgaauugucaugacuagauccacacucauccagguuggcggcuagcccuuc  -3'   obs
    gauuuugugucguuucgcugucgccuugaauggcuguggaucaaguccauguuaagauuucgaauugucaugacuagauccacacucauccagguuggcggcuagcccuuc         exp
    gauuuugugucguuucgcugucgccuugaauggcuguggaucaaguccauguuaagauuucgaauugucaugacuagauccacacucauccagguuggcggcuagcccuuc         known
    (((.....)))(((..(((((((.((((.((((.((((((((.((((.(((.(((.........))).))))))).)))))))).)))).)))).))))))).))).....   reads   mm   sample
    .....uguguUguuucgcugucgccuuga..................................................................................   1       1    seq
    .....uguguUguuucgcugucgccuugaauggcug...........................................................................   1       1    seq
    .......uguUguuucgcugucgccuuga..................................................................................   77      1    seq
    .......ugucguuucgcugucgccuuga..................................................................................   1       0    seq
    .......uguUguuucgcugucgccuugaauggcugug.........................................................................   1       1    seq
    ........guUguuucgcugucgccuuga..................................................................................   2       1    seq
    .........uUguuucgcugucgccuug...................................................................................   1       1    seq
    .........ucguuucgcugucgccuuga..................................................................................   1       0    seq
    .........uUguuucgcugucgccuuga..................................................................................   107     1    seq
    ..........Uguuucgcugucgccuuga..................................................................................   135     1    seq
    ..........Uguuucgcugucgccuugaauggcugug.........................................................................   1       1    seq
    ..........Uguuucgcugucgccuugaauggcugugg........................................................................   1       1    seq
    .........................uugaauggcuguggaucaagu.................................................................   6       0    seq
    .........................uugaauggcuguggaucaaguc................................................................   6       0    seq
    .........................uugaauggcuguggaucaagucA...............................................................   1       1    seq
    .........................uugaauggcuguggaucaagucca..............................................................   1       0    seq
    ..........................ugaauggcuguggaucaagucc...............................................................   2       0    seq
    ..........................ugaauggcuguggaucaagucca..............................................................   14      0    seq
    ..........................Ggaauggcuguggaucaagucca..............................................................   1       1    seq
    ...........................Caauggcuguggaucaaguccaug............................................................   1       1    seq
    ............................aauggcuguggaucaaguc................................................................   7       0    seq
    ............................aauggcuguggaucaagucc...............................................................   16      0    seq
    ............................aauggAuguggaucaagucc...............................................................   1       1    seq
    ............................aauggcuguggauGaagucca..............................................................   1       1    seq
    ............................aauggcugCggaucaagucca..............................................................   1       1    seq
    ............................aauggcuguggUucaagucca..............................................................   1       1    seq
    ............................aauggcuguAgaucaagucca..............................................................   1       1    seq
    ............................aauggcuguggaucaagucca..............................................................   367     0    seq
    ............................aauggcuguggaucaagucUa..............................................................   2       1    seq
    ............................aauggcuguggaucaagucAa..............................................................   1       1    seq
    ............................aauggcuguggaucaaguccaG.............................................................   1       1    seq
    ............................aaugAcuguggaucaaguccau.............................................................   1       1    seq
```

Star **Mature**

```
gauuuugugucguuucgcugucgccuugaauggcuguggaucaaguccauguuaagauuucgaauugucaugacuagauccacacucauccagguuggcggcuagcccuuc
...........................aauggcuguggaucaaguccau..............................................................  407    0  seq
...........................aauggcuguggauUaaguccau..............................................................  1      1  seq
...........................aauggcGguggaucaaguccau..............................................................  1      1  seq
...........................aauggcugAggaucaaguccau..............................................................  2      1  seq
...........................aauggcuguAgaucaaguccau..............................................................  1      1  seq
...........................aauggcuguggaucaagAccaug.............................................................  1      1  seq
...........................aauggcuguggaucaagucAaug.............................................................  4      1  seq
...........................aGuggcuguggaucaaguccaug.............................................................  2      1  seq
...........................aauggcuguAgaucaaguccaug.............................................................  5      1  seq
...........................aauggcuguggaucaaguccauU.............................................................  59     1  seq
...........................aauggcuguggaucaagucGaug.............................................................  1      1  seq
...........................Cauggcuguggaucaaguccaug.............................................................  5      1  seq
...........................aauggcuguggaucaaguccauA.............................................................  28     1  seq
...........................aauggAuguggaucaaguccaug.............................................................  1      1  seq
...........................Gauggcuguggaucaaguccaug.............................................................  1      1  seq
...........................aauggcugCggaucaaguccaug.............................................................  1      1  seq
...........................aauggcuguggaAcaaguccaug.............................................................  1      1  seq
...........................aauggcuguggaucaaguUcaug.............................................................  2      1  seq
...........................Uauggcuguggaucaaguccaug.............................................................  1      1  seq
...........................aauggcuguggaucaaguccaug.............................................................  1811   0  seq
...........................aauggcuguggaucaagucUaug.............................................................  6      1  seq
...........................aauggcuguggUucaaguccaug.............................................................  1      1  seq
...........................aauggcugAggaucaaguccaug.............................................................  2      1  seq
...........................aauggcuguggauUaaguccaug.............................................................  4      1  seq
...........................aauggcuguggauAaaguccaug.............................................................  1      1  seq
...........................aauggcuguggaucaaguccauUu............................................................  1      1  seq
............................auggcuguggaucaaguc.................................................................  36     0  seq
............................auggcuguggaucaagucc................................................................  75     0  seq
............................auggcuguggaucaagucA................................................................  1      1  seq
............................auggcuguggaucaaguUca...............................................................  2      1  seq
............................auggcuguggaucaagucca...............................................................  1062   0  seq
............................augAcuguggaucaagucca...............................................................  1      1  seq
............................auggcuguggaucaaguccU................................................................  1      1  seq
............................auggcAguggaucaagucca...............................................................  1      1  seq
............................auggcuguggaucaaguccG................................................................  3      1  seq
............................auggcugAggaucaagucca...............................................................  1      1  seq
............................aAggcuguggaucaagucca...............................................................  1      1  seq
............................auggcuguAgaucaagucca...............................................................  3      1  seq
............................auggcuCuggaucaagucca...............................................................  1      1  seq
............................auggcuguggauUaagucca...............................................................  1      1  seq
............................Guggcuguggaucaagucca...............................................................  12     1  seq
............................Guggcuguggaucaaguccau..............................................................  6      1  seq
............................auggcuguggaucaaguccaA..............................................................  1      1  seq
............................auggcuguggaucaaguUcau..............................................................  1      1  seq
............................auggcuguAgaucaaguccau..............................................................  2      1  seq
............................auggcuguggaucaaguccau..............................................................  442    0  seq
............................auggcugAggaucaaguccau..............................................................  1      1  seq
............................auggcuguggaucaagucUau..............................................................  1      1  seq
............................auggcAguggaucaaguccau..............................................................  2      1  seq
............................auggcuguggaucaaguccaG..............................................................  1      1  seq
............................augAcuguggaucaaguccaug.............................................................  9      1  seq
............................auggcuguggUucaaguccaug.............................................................  5      1  seq
............................auggcuguggaucaagucUaug.............................................................  53     1  seq
............................auggcuguggaucaUguccaug.............................................................  1      1  seq
............................aNggcuguggaucaaguccaug.............................................................  2      1  seq
............................aGggcuguggaucaaguccaug.............................................................  1      1  seq
............................auggcuguggauUaaguccaug.............................................................  29     1  seq
............................aCggcuguggaucaaguccaug.............................................................  5      1  seq
............................auggcuguggGucaaguccaug.............................................................  2      1  seq
............................auggcuguggCucaaguccaug.............................................................  1      1  seq
............................auggcuguggaucaagCccaug.............................................................  1      1  seq
............................auggcuguggaucaaUuccaug.............................................................  1      1  seq
............................auggcuguggaucaaguccaug.............................................................  26513  0  seq
............................Guggcuguggaucaaguccaug.............................................................  272    1  seq
............................auggcuUuggaucaaguccaug.............................................................  1      1  seq
............................auggcugugAaucaaguccaug.............................................................  5      1  seq
............................auggAuguggaucaaguccaug.............................................................  3      1  seq
............................auggcuguggaAcaaguccaug.............................................................  9      1  seq
............................auggcAguggaucaaguccaug.............................................................  13     1  seq
............................auggcuguggaucaaguAcaug.............................................................  3      1  seq
```

```
                             Star                                    Mature
gauuuugugucguuucgcugucgccuugaauggcuguggaucaaguccauguuaagauuucgaauugucaugacuagauccacacucauccagguuggcggcuagcccuuc
.............................auggcuguggaucaGguccaug............................................................ 3    1    seq
.............................auggUuguggaucaaguccaug............................................................ 3    1    seq
.............................auggcuguggaucaaguccUug............................................................ 2    1    seq
.............................auggcuguggaucaaAuccaug............................................................ 2    1    seq
.............................auggcuguggaucaaguccauA............................................................ 72   1    seq
.............................auggcuguggaucaaguccauU............................................................ 130  1    seq
.............................auggcugAggaucaaguccaug............................................................ 13   1    seq
.............................auCgcuguggaucaaguccaug............................................................ 3    1    seq
.............................auggcuAuggaucaaguccaug............................................................ 2    1    seq
.............................auggcuguggaucaagucGaug............................................................ 3    1    seq
.............................augUcuguggaucaaguccaug............................................................ 4    1    seq
.............................auggcuguggaucaaCuccaug............................................................ 1    1    seq
.............................auggcuguggaGcaaguccaug............................................................ 3    1    seq
.............................Nuggcuguggaucaaguccaug............................................................ 10   1    seq
.............................auggcuguggaucaaguccaGg............................................................ 19   1    seq
.............................auggcuguggaucaagucAaug............................................................ 22   1    seq
.............................auggcuguggaucaaguccauC............................................................ 8    1    seq
.............................auggcuguAgaucaaguccaug............................................................ 123  1    seq
.............................auggcuguggaucaagAccaug............................................................ 12   1    seq
.............................auggcuguUgaucaaguccaug............................................................ 3    1    seq
.............................auggcuguggaucaaguccaAg............................................................ 5    1    seq
.............................auggcugugCaucaaguccaug............................................................ 2    1    seq
.............................auggcuguggaucaaguccaCg............................................................ 2    1    seq
.............................auggcuguggaucaaguUcaug............................................................ 2    1    seq
.............................auggcuguggaucaaguccGug............................................................ 4    1    seq
.............................auggcuguggaucGaguccaug............................................................ 12   1    seq
.............................auggcugugUaucaaguccaug............................................................ 6    1    seq
.............................auggcCguggaucaaguccaug............................................................ 5    1    seq
.............................auUgcuguggaucaaguccaug............................................................ 1    1    seq
.............................Uuggcuguggaucaaguccaug............................................................ 2    1    seq
.............................auggcGguggaucaaguccaug............................................................ 1    1    seq
.............................aAggcuguggaucaaguccaug............................................................ 16   1    seq
.............................auggcuguggauGaaguccaug............................................................ 2    1    seq
.............................auggcuguggauAaaguccaug............................................................ 11   1    seq
.............................auggcuguggaucUaguccaug............................................................ 3    1    seq
.............................auggcuguCgaucaaguccaug............................................................ 2    1    seq
.............................auAgcuguggaucaaguccaug............................................................ 27   1    seq
.............................auggcuguggaucaaguccaugG........................................................... 1    1    seq
.............................auggcuguggaucaaguccaugA........................................................... 17   1    seq
.............................auggcuguggaucaaguccaugu........................................................... 27   0    seq
.............................auAgcuguggaucaaguccaugu........................................................... 1    1    seq
.............................auggcuguggaucaaguccauguu.......................................................... 4    0    seq
.............................auggcuguggaucaaguccauguuaagauu.................................................... 1    0    seq
..............................uggcuguggaucaagucca.............................................................. 1    0    seq
..............................Gggcuguggaucaagucca.............................................................. 1    1    seq
..............................uggcuguggaucaaguccau............................................................. 2    0    seq
..............................uggcuguggaucaaguccauU............................................................ 2    1    seq
..............................Aggcuguggaucaaguccaug............................................................ 1    1    seq
..............................uggcuguggaucaaguccaug............................................................ 88   0    seq
..............................uggcuguggaucaaguccauA............................................................ 1    1    seq
..............................uggcuguggaucaagucAaugu........................................................... 1    1    seq
..............................uggcuguggaucaaguccaugu........................................................... 18   0    seq
..............................uggcuguggaucaaguccauguu.......................................................... 6    0    seq
................................gcuguggaucaaguccaug............................................................ 1    0    seq
.................................cuguggaucaaguccaug............................................................ 1    0    seq
.............................................uccauguuaagauuucgaauugucaugacu.................................... 1    0    seq
.................................................uguuaagauuucgaauugucaugacuagauc............................... 3    0    seq
...................................................uuaagauuucgaauuguc.......................................... 1    0    seq
...................................................uuaagauuucAaauuguca......................................... 1    1    seq
...................................................Auaagauuucgaauuguca......................................... 1    1    seq
...................................................uuaagauuucgaauuguca......................................... 26   0    seq
...................................................uuaagauuucgaauugucau........................................ 1    0    seq
...................................................uuaagauuucgaauugucauU....................................... 4    1    seq
...................................................uuaagauuucgaauugucaugacu.................................... 6    0    seq
...................................................uuaagauuucgaauugucaugacuaga................................. 2    0    seq
...................................................uuaagauuucgaauugucaugacuagaucca............................. 4    0    seq
....................................................uaagauuucgaauugucaugacuagaucca............................. 7    0    seq
....................................................uaagauuucgaauugucaugacuagauccac............................ 1    0    seq
.........................................................uuucgaauugucaugacuagauccacacuca....................... 2    0    seq
..........................................................uucgaauugucaugacuaga................................. 1    0    seq
```

```
                              Star                                  Mature
gauuuugugucguuucgcugucgccuugaauggcuguggaucaaguccauguuaagauuucgaauugucaugacuagauccacacucauccagguuggcggcuagcccuuc
..........................................................uucgaauugucaugacuagauccacacu.........................   1      0   seq
..........................................................uucgaauugucaugacuagauccacacuca.......................   10     0   seq
..........................................................uucgaauugAcaugacuagauccacacuca.......................   1      1   seq
..........................................................uucgaauugucaugacuagauccacacucau.....................   6      0   seq
...........................................................ucgaauugucaugacuGgaucca.............................   1      1   seq
...........................................................ucgaauugucaugacuagauccacacucauc.....................   1      0   seq
................................................................uugucaugacuagauccacacu.........................   97     0   seq
................................................................uugucaugacuagauccacacucauccagg................   1      0   seq
.................................................................ugucaugacuagauccacacucauccagg................   1      0   seq
.................................................................ugucaugacuagauccacacucauccaggu...............   4      0   seq
.................................................................ugucaugacuagauccacacucauccagguu..............   1      0   seq
..................................................................gucaugacuagauccacacuc........................   1      0   seq
..................................................................gucaugacuagauccacacuca.......................   9      0   seq
..................................................................gucaugacuagauccacacucUu......................   1      1   seq
...................................................................ucaugacuagauccacacucau......................   1      0   seq
....................................................................cCugacuagauccacacu.........................   63     1   seq
....................................................................caugacuagauccacacucau......................   2      0   seq
....................................................................Aaugacuagauccacacucaucc....................   1      1   seq
....................................................................caugacuagauccacacucaucc....................   2      0   seq
.....................................................................Cugacuagauccacacuca.......................   1      1   seq
.....................................................................augacuagauccacacucau......................   24     0   seq
.....................................................................Cugacuagauccacacucau......................   16     1   seq
.....................................................................Uugacuagauccacacucau......................   2      1   seq
.....................................................................Cugacuagauccacacucauc.....................   8      1   seq
.....................................................................augacuagauccacacucauc.....................   13     0   seq
.....................................................................Cugacuagauccacacucaucc....................   258    1   seq
.....................................................................augGcuagauccacacucaucc....................   1      1   seq
.....................................................................augacuagauccacacucaucc....................   156    0   seq
.....................................................................augacuagauccacacAcaucc....................   1      1   seq
.....................................................................Uugacuagauccacacucaucc....................   6      1   seq
.....................................................................augacuagauccacacucaucU....................   1      1   seq
.....................................................................augacuagauAcacacucaucc....................   1      1   seq
.....................................................................Cugacuagauccacacucaucca...................   1      1   seq
......................................................................ugacuagauccUcacuca.......................   1      1   seq
......................................................................ugacuagauccacaUuca.......................   1      1   seq
......................................................................uAacuagauccacacuca.......................   2      1   seq
......................................................................ugacuagauccacacuGa.......................   3      1   seq
......................................................................ugacAagauccacacuca.......................   8      1   seq
......................................................................ugacuagauccacacuca.......................   6176   0   seq
......................................................................ugacuagauccCcacuca.......................   1      1   seq
......................................................................ugacuagauccacacuUa.......................   7      1   seq
......................................................................ugacuaUauccacacuca.......................   2      1   seq
......................................................................ugaUuagauccacacuca.......................   1      1   seq
......................................................................ugacuGgauccacacuca.......................   2      1   seq
......................................................................ugacuagauccacacAca.......................   2      1   seq
......................................................................Agacuagauccacacuca.......................   30     1   seq
......................................................................ugacuaAauccacacuca.......................   2      1   seq
......................................................................ugacuagauccacacucG.......................   5      1   seq
......................................................................ugacuagaucUacacuca.......................   3      1   seq
......................................................................ugacuagaAccacacuca.......................   1      1   seq
......................................................................ugacuagauccacacuAa.......................   1      1   seq
......................................................................ugacuagaucNacacuca.......................   1      1   seq
......................................................................Cgacuagauccacacuca.......................   1      1   seq
......................................................................ugacuagauccaAacuca.......................   1      1   seq
......................................................................ugacuagaucAacacuca.......................   1      1   seq
......................................................................ugacuagUuccacacuca.......................   1      1   seq
......................................................................ugacuagauccGcacuca.......................   3      1   seq
......................................................................ugacuagauAcacacuca.......................   12     1   seq
......................................................................ugacuagauccacGcuca.......................   2      1   seq
......................................................................Ngacuagauccacacuca.......................   2      1   seq
......................................................................ugacuagGuccacacuca.......................   2      1   seq
......................................................................ugGcuagauccacacuca.......................   2      1   seq
......................................................................ugacCagauccacacuca.......................   1      1   seq
......................................................................ugacuagauccacaAuca.......................   4      1   seq
......................................................................Ggacuagauccacacuca.......................   6      1   seq
......................................................................ugacuagauccacCcuca.......................   1      1   seq
......................................................................ugacuagauccaGacuca.......................   1      1   seq
......................................................................ugacuagauccUcacucau......................   30     1   seq
......................................................................ugacuagauccacUcucau......................   32     1   seq
......................................................................ugacuagauccCcacucau......................   1      1   seq
```

Star Mature

```
gauuuugugucguuucgcugucgccuugaauggcuguggaucaaguccauguuaagauuucgaauugucaugacuagauccacacucauccagguuggcggcuagcccuuc
.....................................................................uUacuagauccacacucau.......................   23      1   seq
.....................................................................ugacuagauccacCcucau.......................   5       1   seq
.....................................................................ugacuagauccGcacucau.......................   28      1   seq
.....................................................................ugaUuagauccacacucau.......................   16      1   seq
.....................................................................ugacuagaAccacacucau.......................   43      1   seq
.....................................................................ugacuagauccacacucaC.......................   12      1   seq
.....................................................................ugacuagauccacaAucau.......................   9       1   seq
.....................................................................ugacuagaGccacacucau.......................   8       1   seq
.....................................................................Ngacuagauccacacucau.......................   50      1   seq
.....................................................................ugacuagaucGacacucau.......................   20      1   seq
.....................................................................ugGcuagauccacacucau.......................   26      1   seq
.....................................................................Agacuagauccacacucau.......................   687     1   seq
.....................................................................ugaGuagauccacacucau.......................   11      1   seq
.....................................................................ugacuagaucAacacucau.......................   69      1   seq
.....................................................................ugacAagauccacacucau.......................   181     1   seq
.....................................................................ugacuagauUcacacucau.......................   43      1   seq
.....................................................................ugacuagauccacacucUu.......................   16      1   seq
.....................................................................ugUcuagauccacacucau.......................   23      1   seq
.....................................................................ugaAuagauccacacucau.......................   14      1   seq
.....................................................................ugacuagaucUacacucau.......................   177     1   seq
.....................................................................ugacuagauccacacucau.......................   175059  0   seq
.....................................................................ugacuagauccaGacucau.......................   11      1   seq
.....................................................................ugacuagauGcacacucau.......................   10      1   seq
.....................................................................ugacuagauccaAacucau.......................   30      1   seq
.....................................................................ugacuagaCccacacucau.......................   24      1   seq
.....................................................................ugCcuagauccacacucau.......................   2       1   seq
.....................................................................ugacuagauccacaUucau.......................   5       1   seq
.....................................................................Ggacuagauccacacucau.......................   90      1   seq
.....................................................................ugacuCgauccacacucau.......................   1       1   seq
.....................................................................ugacuagauccacacucaA.......................   89      1   seq
.....................................................................ugacuagCuccacacucau.......................   6       1   seq
.....................................................................ugacuagauccaUacucau.......................   24      1   seq
.....................................................................ugacuagUuccacacucau.......................   18      1   seq
.....................................................................ugacuagauAcacacucau.......................   230     1   seq
.....................................................................ugacuUgauccacacucau.......................   20      1   seq
.....................................................................ugacuagauccacacuUau.......................   130     1   seq
.....................................................................ugacuaAauccacacucau.......................   36      1   seq
.....................................................................ugacuGgauccacacucau.......................   105     1   seq
.....................................................................ugacuagauccacacuAau.......................   29      1   seq
.....................................................................ugacCagauccacacucau.......................   20      1   seq
.....................................................................ugacuagauccacacCcau.......................   17      1   seq
.....................................................................uAacuagauccacacucau.......................   72      1   seq
.....................................................................ugacGagauccacacucau.......................   11      1   seq
.....................................................................ugacuagauccacacuGau.......................   8       1   seq
.....................................................................ugacuagauccacGcucau.......................   32      1   seq
.....................................................................ugacuNgauccacacucau.......................   3       1   seq
.....................................................................ugacuagauccacacGcau.......................   6       1   seq
.....................................................................uNacuagauccacacucau.......................   6       1   seq
.....................................................................ugacuagauccacacAcau.......................   99      1   seq
.....................................................................ugacuagauccacacucGu.......................   36      1   seq
.....................................................................Cgacuagauccacacucau.......................   34      1   seq
.....................................................................uCacuagauccacacucau.......................   16      1   seq
.....................................................................ugacuagauccacacucaG.......................   76      1   seq
.....................................................................ugacuagauccacacucCu.......................   1       1   seq
.....................................................................ugacuaUauccacacucau.......................   16      1   seq
.....................................................................ugacuagauccacaGucau.......................   5       1   seq
.....................................................................ugacuagGuccacacucau.......................   28      1   seq
.....................................................................Cgacuagauccacacucauc......................   7       1   seq
.....................................................................ugacuagaGccacacucauc......................   3       1   seq
.....................................................................ugacuGgauccacacucauc......................   14      1   seq
.....................................................................ugaUuagauccacacucauc......................   4       1   seq
.....................................................................ugacuagauccacacuAauc......................   12      1   seq
.....................................................................ugaAuagauccacacucauc......................   6       1   seq
.....................................................................ugacuagUuccacacucauc......................   6       1   seq
.....................................................................ugacuagauccacacucaAc......................   11      1   seq
.....................................................................ugacuagauccacacucauG......................   3       1   seq
.....................................................................uUacuagauccacacucauc......................   7       1   seq
.....................................................................ugacuagaAccacacucauc......................   11      1   seq
.....................................................................ugacuagauccCcacucauc......................   1       1   seq
.....................................................................ugacuagauccacNcucauc......................   1       1   seq
```

```
                                 Star                                         Mature
gauuuugugucguuucgcugucgccuugaauggcuguggaucaaguccauguuaagauuucgaauugucaugacuagauccacacucauccagguuggcggcuagcccuuc
......................................................................ugacuagauccacGcucauc.....................   10      1   seq
......................................................................uAacuagauccacacucauc.....................   22      1   seq
......................................................................ugacuagauccacacucauU.....................   885     1   seq
......................................................................ugacuagauccGcacucauc.....................   11      1   seq
......................................................................ugacuagaCccacacucauc.....................   3       1   seq
......................................................................ugacuagauccacUcucauc.....................   6       1   seq
......................................................................ugacuagauccacacucCuc.....................   1       1   seq
......................................................................ugacAagauccacacucauc.....................   48      1   seq
......................................................................ugacuagauccacacGcauc.....................   2       1   seq
......................................................................ugacuUgauccacacucauc.....................   5       1   seq
......................................................................ugacuagauAcacacucauc.....................   67      1   seq
......................................................................Agacuagauccacacucauc.....................   176     1   seq
......................................................................ugacuagauUcacacucauc.....................   15      1   seq
......................................................................ugacuagauccacCcucauc.....................   1       1   seq
......................................................................Ggacuagauccacacucauc.....................   25      1   seq
......................................................................ugacuagaucUacacucauc.....................   41      1   seq
......................................................................ugacuagauccacacAcauc.....................   24      1   seq
......................................................................ugacuagGuccacacucauc.....................   11      1   seq
......................................................................ugacuagauccacaUucauc.....................   4       1   seq
......................................................................ugUcuagauccacacucauc.....................   7       1   seq
......................................................................ugacuagauccUcacucauc.....................   8       1   seq
......................................................................ugacuagauccacacuUauc.....................   26      1   seq
......................................................................ugacuagaucAacacucauc.....................   13      1   seq
......................................................................ugacuagauccacacucauA.....................   531     1   seq
......................................................................ugacuagauccacacucGuc.....................   18      1   seq
......................................................................ugacuagauccacacucaCc.....................   11      1   seq
......................................................................ugCcuagauccacacucauc.....................   2       1   seq
......................................................................ugacuagauGcacacucauc.....................   1       1   seq
......................................................................ugacuagauccaGacucauc.....................   6       1   seq
......................................................................ugGcuagauccacacucauc.....................   6       1   seq
......................................................................ugacuagauccacacCcauc.....................   7       1   seq
......................................................................ugacuagCuccacacucauc.....................   3       1   seq
......................................................................ugacuagauccacacucaGc.....................   2       1   seq
......................................................................ugacuagauccacacucauc.....................   50870   0   seq
......................................................................ugacuagauccacaAucauc.....................   6       1   seq
......................................................................uCacuagauccacacucauc.....................   5       1   seq
......................................................................ugacuNgauccacacucauc.....................   1       1   seq
......................................................................ugacuaAauccacacucauc.....................   17      1   seq
......................................................................ugacuaCauccacacucauc.....................   2       1   seq
......................................................................ugaGuagauccacacucauc.....................   4       1   seq
......................................................................ugacuaUauccacacucauc.....................   6       1   seq
......................................................................ugacuagauccacacucUuc.....................   9       1   seq
......................................................................Ngacuagauccacacucauc.....................   11      1   seq
......................................................................ugacCagauccacacucauc.....................   5       1   seq
......................................................................ugacuagauccaUacucauc.....................   10      1   seq
......................................................................ugacuagauccaAacucauc.....................   6       1   seq
......................................................................ugacuagaucGacacucauc.....................   4       1   seq
......................................................................ugacuagaucUacacucaucc....................   600     1   seq
......................................................................ugacuCgauccacacucaucc....................   8       1   seq
......................................................................ugacuagauccUcacucaucc....................   60      1   seq
......................................................................ugacuagaCccacacucaucc....................   39      1   seq
......................................................................ugacCagauccacacucaucc....................   55      1   seq
......................................................................ugacuagauccacacGcaucc....................   12      1   seq
......................................................................ugacuaUauccacacucaucc....................   53      1   seq
......................................................................ugacAagauccacacucaucc....................   399     1   seq
......................................................................ugacuagauccaUacucaucc....................   58      1   seq
......................................................................ugacuagauGcacacucaucc....................   11      1   seq
......................................................................ugacuagaGccacacucaucc....................   14      1   seq
......................................................................ugaGuagauccacacucaucc....................   18      1   seq
......................................................................ugacuaAauccacacucaucc....................   150     1   seq
......................................................................ugacuagaucGacacucaucc....................   34      1   seq
......................................................................ugacGagauccacacucaucc....................   20      1   seq
......................................................................uNacuagauccacacucaucc....................   14      1   seq
......................................................................ugacuagauccacacucUucc....................   34      1   seq
......................................................................Agacuagauccacacucaucc....................   1604    1   seq
......................................................................ugacuagGuccacacucaucc....................   49      1   seq
......................................................................ugacuagauUcacacucaucc....................   97      1   seq
......................................................................ugacuagauccacacucauUc....................   88      1   seq
......................................................................ugacuUgauccacacucaucc....................   40      1   seq
......................................................................ugacuagauccacacucaCcc....................   52      1   seq
```

Star Mature

```
gauuuugugucguuucgcugucgccuugaauggcuguggaucaaguccauguuaagauuucgaauugucaugacuagauccacacucauccagguuggcggcuagcccuuc
......................................................................ugacuagauccacacucauNc....................	1	1	seq
......................................................................ugacuagauccacacAcaucc....................	227	1	seq
......................................................................ugacuagauccacacucaNcc....................	1	1	seq
......................................................................ugacuagauccacacucauGc....................	12	1	seq
......................................................................ugCcuagauccacacucaucc....................	6	1	seq
......................................................................ugacuagauccacacucaucG....................	65	1	seq
......................................................................ugacuagauccacacuGaucc....................	21	1	seq
......................................................................ugacuagauccacaAucaucc....................	32	1	seq
......................................................................ugacuagCuccacacucaucc....................	14	1	seq
......................................................................ugacuagauccaGacucaucc....................	42	1	seq
......................................................................ugacuagauccacGcucaucc....................	66	1	seq
......................................................................ugacuagaucAacacucaucc....................	158	1	seq
......................................................................ugacuagauccaAacucaucc....................	69	1	seq
......................................................................uUacuagauccacacucaucc....................	33	1	seq
......................................................................ugacuGgauccacacucaucc....................	254	1	seq
......................................................................ugacuagauccacacucaucc....................	435681	0	seq
......................................................................ugUcuagauccacacucaucc....................	44	1	seq
......................................................................Ngacuagauccacacucaucc....................	140	1	seq
......................................................................ugacuagauccacacucGucc....................	111	1	seq
......................................................................Cgacuagauccacacucaucc....................	96	1	seq
......................................................................ugGcuagauccacacucaucc....................	64	1	seq
......................................................................ugacuagauccacacuAaucc....................	74	1	seq
......................................................................ugacuagauccacaUucaucc....................	40	1	seq
......................................................................ugNcuagauccacacucaucc....................	3	1	seq
......................................................................ugacuagaAccacacucaucc....................	118	1	seq
......................................................................ugacuagauccacacuUaucc....................	321	1	seq
......................................................................ugacuagauccacacucaucU....................	6060	1	seq
......................................................................ugacuagauccacCcucaucc....................	26	1	seq
......................................................................ugacuagauccacacucaucN....................	1	1	seq
......................................................................ugacuagauccGcacucaucc....................	71	1	seq
......................................................................ugacuagauccacacucaGcc....................	7	1	seq
......................................................................ugacuagauccacacucaucA....................	176	1	seq
......................................................................ugacuagauccacacucauAc....................	374	1	seq
......................................................................Ggacuagauccacacucaucc....................	254	1	seq
......................................................................ugacuagauccacUcucaucc....................	72	1	seq
......................................................................ugacuagauccacacucaAcc....................	164	1	seq
......................................................................uCacuagauccacacucaucc....................	57	1	seq
......................................................................ugacuNgauccacacucaucc....................	3	1	seq
......................................................................ugaAuagauccacacucaucc....................	60	1	seq
......................................................................ugaUuagauccacacucaucc....................	36	1	seq
......................................................................ugacuaCauccacacucaucc....................	13	1	seq
......................................................................ugacuagUuccacacucaucc....................	54	1	seq
......................................................................ugacuagauccacacucCucc....................	10	1	seq
......................................................................ugacuagauAcacacucaucc....................	548	1	seq
......................................................................ugacuagauccCcacucaucc....................	6	1	seq
......................................................................uAacuagauccacacucaucc....................	228	1	seq
......................................................................ugacuagauccacacCcaucc....................	33	1	seq
......................................................................ugacuagauccacaGucaucc....................	15	1	seq
......................................................................ugacuagGuccacacucaucca...................	1	1	seq
......................................................................ugCcuagauccacacucaucca...................	1	1	seq
......................................................................ugacuCgauccacacucaucca...................	1	1	seq
......................................................................ugacuagauccacacucaucAa...................	2	1	seq
......................................................................ugacuagauccacacucauccC...................	89	1	seq
......................................................................ugacuaAauccacacucaucca...................	2	1	seq
......................................................................ugacuagauccacGcucaucca...................	1	1	seq
......................................................................ugacuagaucUacacucaucca...................	3	1	seq
......................................................................ugacuagaucGacacucaucca...................	1	1	seq
......................................................................ugacuagauccacacucauAca...................	2	1	seq
......................................................................Agacuagauccacacucaucca...................	9	1	seq
......................................................................ugacuagauccacaGucaucca...................	1	1	seq
......................................................................ugacuagauAcacacucaucca...................	5	1	seq
......................................................................Ggacuagauccacacucaucca...................	2	1	seq
......................................................................uCacuagauccacacucaucca...................	2	1	seq
......................................................................ugacAagauccacacucaucca...................	2	1	seq
......................................................................ugacuaUauccacacucaucca...................	2	1	seq
......................................................................ugacuagauccacacucauccG...................	91	1	seq
......................................................................ugacuagauccacacucaucca...................	2602	0	seq
......................................................................ugacuagauccacaAucaucca...................	1	1	seq
......................................................................ugacuagauccacacucaucUa...................	4	1	seq
......................................................................uAacuagauccacacucaucca...................	3	1	seq
```

```
                             Star                                     Mature
gauuuugugucguuucgcugucgccuugaauggcuguggaucaaguccauguuaagauuucgaauugucaugacuagauccacacucauccagguuggcggcuagcccuuc
......................................................................ugacuagaGccacacucaucca...................    1          1          seq
......................................................................ugacuagauccacacucauccU...................    23162      1          seq
......................................................................ugacuagaucAacacucaucca...................    2          1          seq
......................................................................ugacuagauccacacucaAcca...................    2          1          seq
......................................................................ugacuagauccacacucauccaA..................    25         1          seq
......................................................................ugacuagauccacacucauccaU..................    54         1          seq
......................................................................ugacuagauccacacucauccCg..................    1          1          seq
......................................................................ugacuagauccacacucauccag..................    1          0          seq
......................................................................ugacuagauccacacucauccagg.................    1          0          seq
.......................................................................Uacuagauccacacucau......................    7          1          seq
.......................................................................gacAagauccacacucau......................    2          1          seq
.......................................................................gacuagauAcacacucau......................    1          1          seq
.......................................................................gacuGgauccacacucau......................    3          1          seq
.......................................................................gacuagauccacacucaA......................    1          1          seq
.......................................................................gGcuagauccacacucau......................    2          1          seq
.......................................................................gacuagauccacacucau......................    423        0          seq
.......................................................................gacuagauccacacAcau......................    1          1          seq
.......................................................................gacuagaucUacacucau......................    2          1          seq
.......................................................................gacuagaucAacacucau......................    1          1          seq
.......................................................................gacuaUauccacacucau......................    1          1          seq
.......................................................................gGcuagauccacacucauc.....................    2          1          seq
.......................................................................gacuagauccacacucauc.....................    284        0          seq
.......................................................................gacuagauAcacacucauc.....................    1          1          seq
.......................................................................gacuagauccacacucUuc.....................    1          1          seq
.......................................................................gacuagaCccacacucauc.....................    1          1          seq
.......................................................................gacuagauccacacucGucc....................    1          1          seq
.......................................................................Uacuagauccacacucaucc....................    5          1          seq
.......................................................................Nacuagauccacacucaucc....................    1          1          seq
.......................................................................gacuagauccacacuAaucc....................    1          1          seq
.......................................................................gGcuagauccacacucaucc....................    7          1          seq
.......................................................................gacuagCuccacacucaucc....................    1          1          seq
.......................................................................gacuagauccacacucaucA....................    3          1          seq
.......................................................................gacuagauccacacucaucc....................    1025       0          seq
.......................................................................Aacuagauccacacucaucc....................    1          1          seq
.......................................................................gacuagauccacacucauAc....................    2          1          seq
.......................................................................gacuagauAcacacucaucc....................    2          1          seq
.......................................................................gacuagauccacacAcaucc....................    4          1          seq
.......................................................................gacuGgauccacacucaucc....................    13         1          seq
.......................................................................gacuagauUcacacucaucc....................    1          1          seq
.......................................................................gacuagaAccacacucaucc....................    1          1          seq
.......................................................................gacAagauccacacucaucc....................    6          1          seq
.......................................................................gacuagauccacacuUaucc....................    1          1          seq
.......................................................................gacuagauccacacucaucU....................    14         1          seq
.......................................................................gacuagaucAacacucaucc....................    2          1          seq
.......................................................................gacuagauccacacucauccU...................    197        1          seq
.......................................................................gacuagauccacacucauccC...................    1          1          seq
.......................................................................gacuagauccacacucaucca...................    3          0          seq
........................................................................acuagauccacacucaucc....................    8          0          seq
........................................................................acuagauccacacucauccagg.................    1          0          seq
.........................................................................cuagauccacacucaucc....................    11         0          seq
.........................................................................cuagauccacacucauccaggu................    1          0          seq
..........................................................................uagauccacacucauccaggu................    1          0          seq
.....................................................................................ucauccagguuggcggcuagcccuu.    1          0          seq
...........................................................................................agguuggcggcuagcccuuc    1          0          seq
```
